# Thermodynamics of RTA3 peptide binding to membranes and consequences for antimicrobial activity^☆^

**DOI:** 10.1016/j.bbamem.2010.03.017

**Published:** 2010-06

**Authors:** Ayman Hawrani, Robin A. Howe, Timothy R. Walsh, Christopher E. Dempsey

**Affiliations:** a Department of Cellular and Molecular Medicine, Bristol University, Bristol, BS8 1TD, UK; b Department of Medical Microbiology, Cardiff University, Cardiff CF14 4XN, UK; c NPHS Cardiff, University Hospital of Wales, Cardiff CF14 4XW, UK; d Biochemistry Department, Bristol University, Bristol, BS8 1TD, UK

**Keywords:** buffer A, 10 mM Tris–HCl, 107 mM NaCl, pH 7.4, CD, circular dichroism, CF, carboxyfluorescein, CH, cholesterol, CL, cardiolipin, POPC, palmitoyloleoylphosphatidylcholine, FIC, fractional inhibitory concentration, FPE, fluorescein phosphatidylethanolamine, IM, inner membrane, LUV, large unilamellar vesicle, MIC, minimum inhibitory concentration, OM, outer membrane, ONPG, ortho-nitrophenyl-β-galactoside, PC, phosphatidylcholine, PE, phosphatidylethanolamine, PG, phosphatidylglycerol, Amphipathic peptide, Interfacial binding, Cysteine, Free energy, Commensal organism, Phospholipid bilayer

## Abstract

RTA3 is an α-helical, amphipathic peptide with broad-spectrum activity against Gram-negative bacteria and low mammalian cell toxicity. RTA3 contains a cysteine residue, replacement of which with an alanine or serine (RTA3-C15S) virtually abolishes antimicrobial activity. Much of the activity of RTA3 can be recovered in RTA3-C15L, indicating that the C15 residue functions largely as a bulky hydrophobic side chain promoting target cell membrane interactions. The poorly active RTA3-C15S is a useful variant for assessing the mechanistic aspects of RTA3 activity. Binding and membrane perturbation in vesicles containing different proportions of negative surface charge are analyzed in terms of amino acid-specific free energy contributions to interfacial binding, which likely underlie variations in antimicrobial activity amongst RTA3 variants. Comparison with published free energy scales indicates that the reduced electrostatic contribution to binding to membranes having reduced negative surface charge can be compensated in RTA3 (but not RTA3-C15S) by a slightly deeper insertion of the C-terminus of the peptide to maximize hydrophobic contributions to binding. Analysis of inner membrane (IM)- and outer membrane (OM)-selective permeabilization of *Escherichiacoli* demonstrates a broad similarity between peptide effects on vesicles with low negative surface charge (20% negatively charged lipids), *E.coli* membrane perturbation, and antimicrobial activity, supporting a role for membrane perturbation in the killing mechanism of RTA3. The results demonstrate that large variations in antimicrobial activity on subtle changes in amino acid sequence in helical amphipathic peptides can be rationalized in terms of the thermodynamics of peptide binding to membranes, allowing a more systematic understanding of antimicrobial activity in these peptides.

## 1 Introduction

The antimicrobial peptide RTA3, an optimized version of a commensal-derived peptide, has strong anti-Gram-negative activity combined with low mammalian cell toxicity [1]. RTA3 is a C-terminally amidated 16 residue peptide rich in basic amino acids (Arg and Lys; see amino acid sequence in Table 1) and conforms to the positively charged amphipathic helical motif of a large class of antimicrobial peptides [2]. These peptides are widely distributed in nature and have been isolated from a variety of sources including mammalian tissues, amphibia, insects, bacteria, and plants [2–6]. Amphipathic peptides are bactericidal and fast acting compared to conventional antibiotics, with a broad spectrum of activities against targets such as bacteria, fungi, viruses, and tumour cell lines. Therapeutic potential in these peptides resides in selective killing of target bacteria over normal mammalian cells, with this selectivity arising largely from the minimal negative surface charge in the external bilayer leaflet of mammalian cells, compared with the high content of negatively charged lipids in bacterial cell membranes which greatly promotes binding of positively charged peptides [7,8].

We recently showed that the low mammalian cell toxicity of RTA3 results from disruption of the nonpolar helix face of the peptide with an arginine (R5) residue which abolishes binding to neutral membranes such as those of mammalian cells [1]. The positive charge is accommodated in negatively charged membranes, possibly by tilting of the peptide helix at the membrane interface to allow the arginine side chain to access the well-solvated polar regions of the membrane. RTA3 also contains a cysteine residue, replacement of which in an alanine-scan mutagenesis study greatly reduces antimicrobial activity. Cysteine residues have a variety of roles in antimicrobial peptides. In some cases, such as mammalian cryptidins [9], or tree frog distinctins [10], cysteine residues are involved in homo- or heterodimerization. Some antimicrobial peptides have internal disulfide bonds [11]. In other cases (e.g., maximins from frog skin [12]), antimicrobial activity requires a free cysteine thiol. The latter observation may suggest a role for a free cysteine residue in receptor-mediated heterodisulphide interactions or perhaps in metal ion binding.

Here, we assess the mechanism of action of RTA3, focussing on the role of the C15 residue. In the first part of the paper, we characterise the relationship between membrane-specific perturbation and antimicrobial activity and show that peptide binding to membranes and membrane perturbation can be understood in terms of free energy contributions to the partitioning of helical peptides into membranes. In the second part, we characterise *Escherichia* *coli* inner and outer membrane-specific perturbation by the peptides and assess their relationships to bacterial killing. We find that a Cys15 to Ser15 variant (RTA3-C15S) is useful in addressing mechanistic aspects of the peptide. The data demonstrate that rather subtle structural changes in RTA3 peptides can have large effects on antimicrobial activity and that these can be understood in terms of the thermodynamics of membrane interactions.

## 2 Materials and methods

### 2.1 Peptide synthesis, purification, and characterization

The peptides listed in Table 1 were synthesised by Dr. G. Bloomberg of the Bristol Centre for Molecular Recognition using standard Fmoc solid-phase synthesis. The peptides were purified by HPLC and were confirmed to be at least 97% pure by analytical HPLC and to have the predicted m/e ratio by mass spectrometry. Phospholipids, produced from egg yolk, were from Lipid Products (Nutfield, UK), carboxyfluorescein (CF) was from Sigma (Poole, UK), and fluorescein–phosphatidylethanolamine (FPE) was from Avanti (Alabaster, AL, USA).

### 2.2 Biological activities

Minimum inhibitory concentrations (MIC) of the peptides were determined by broth microdilution according to the Clinical and Laboratory Standards Institute [13]. About 90 µL of 0.5–1 × 10^6^ CFU/mL of *Pseudomonas* *aeruginosa* ATCC 27853 and *E. coli* ATCC 25922 in Mueller Hinton media (plus cations) broth (BD, Baltimore, MD, USA) was incubated in 96-well microtitre plates with serial twofold dilutions of the peptides. Minimum inhibitory concentrations (MICs) were defined as the lowest peptide concentration with no visible growth of bacteria after 24 hours at 37 °C. All measurements were made in triplicate and averaged.

### 2.3 Preparation of lipid vesicles

All experiments were performed at room temperature. Large unilamellar vesicles (100 nm in diameter) were used for all spectroscopic measurements except for circular dichroism (CD) spectroscopy for which smaller (50 nm) vesicles were used to minimize light scattering effects. Lipids were dried from chloroform/methanol solution and pumped under high vacuum to remove traces of solvent. Dried lipids were hydrated at a concentration of 10 mg/mL in 10 mM Tris–HCl, pH 7.4 containing either 107 mM NaCl (buffer A), or, for the CF-dye-release experiments, 50 mM CF. Vesicles doped with FPE were prepared similarly except that 0.5 mol.% of FPE in methanol was added to the lipids in organic solvent before drying. Hydrated lipids were freeze-thawed 3 times and extruded 10 times through two 100-nm or 50-nm pore membranes using a Lipex Biomolecular extruder (Vancouver, Canada). Vesicles for peptide binding, monitored using either tryptophan fluorescence or FPE fluorescence, were used directly. Vesicles for CF-dye-release measurements were used after gel filtration on a Sephadex G-15 column with buffer A as the mobile phase to remove nontrapped CF. Thus, in all experiments, interaction of the peptide with vesicles was determined in the same buffer (buffer A).

### 2.4 Fluorescence and circular dichroism spectroscopy

Fluorescence measurements were made using a SPEX Fluoromax fluorimeter (Horiba Jobin Yvon, Longjumeau, France). Peptide solutions were made in plastic tubes or cuvettes to minimize loss of peptide at low concentrations due to binding to glass surfaces. For the measurement of vesicle-induced changes in the emission spectra of tryptophan in Trp-containing peptides, a 2-µM peptide solution was incubated in buffer A, and aliquots of vesicles suspension were added to give total lipid concentrations in the range 0 to 300 µM total lipid. Tryptophan fluorescence was excited at 280 nm, and the emission spectrum was measured between 300 and 450 nm in 1-nm increments with 1-s signal averaging. We used the lipid-induced spectral blue shift (Δ*λ*, corresponding to the change in wavelength of the emission signal maximum compared to the lipid-free spectrum) to monitor peptide binding and determined using a solution of 2 μM tryptophan in buffer A that light scattering artifacts [14] were insignificant at the lipid concentrations required to sample the binding curves. The value of Δ*λ* was plotted against the lipid concentration and fitted to a simple hyperbolic function (Eq. (1)) to obtain estimates of the maximum fluorescence emission blue shift (∆*λ*_max_) and an apparent binding constant that is equivalent to the concentration of lipid at which the lipid-induced blue shift was half-maximal (*K*_L_).

(1) $$\Delta \lambda \;=\Delta \lambda_{\text{max}}\left[\text{lipid}\right]/\left(K_{\text{L}}\;+\;\left[\text{lipid}\right]\right)$$

An equivalent analysis was made for the FPE binding data. In this case, a vesicle sample in buffer A containing lipid at a total concentration of 65 μM was titrated with aliquots of peptide in the range 0–40 μM while monitoring the fluorescence emission at 520 nm resulting from excitation of FPE electronic transitions at 490 nm. Addition of positive charge to the peptide surface upon peptide binding results in an enhancement of the FPE fluorescence, Δ*F* [15]. In these analyses, Δ*F* (fluorescence intensity minus the peptide-free fluorescence intensity) was plotted against the peptide concentration and fitted to Eq. (2) to obtain estimates of the maximum fluorescence enhancement (Δ*F*_max_) and *K*_P_, the apparent binding constant, equivalent in this case to the peptide concentration at which the peptide-induced fluorescence enhancement was half-maximal.

(2) $$\Delta F\;=\;\Delta F_{\text{max}}\left[\text{peptide}\right]/\left(K_{\text{P}}\;+\;\left[\text{peptide}\right]\right)$$

We note that *K*_L_ and *K*_P_ are not equivalent (i.e., the FPE and Trp fluorescence data cannot be fitted to a single equilibrium binding constant) since the peptide–membrane interaction corresponds to a partitioning rather than a discrete site-binding phenomenon, as described in more detail elsewhere [1]. However, each of these can be used to determine a free energy change for membrane binding, δΔ*G*, that results from changing the peptide sequence, according to Eq. (3), where the subscripts refer to the *K* values for peptides 1 (e.g., wild type) and 2 (single amino acid substitution), determined either from lipid FPE fluorescence (*K*_P_) or peptide Trp fluorescence (*K*_L_).

(3) $$\delta \Delta G\;=\;-\text{RT ln}\left(K_{\text{P1}}/K_{\text{P2}}\right)\text{;}\;\delta \Delta G\;=\;-\text{RTln}\left(K_{\text{L1}}/K_{\text{L2}}\right)$$

Peptide-induced dye release from vesicles loaded with CF was measured from the loss of CF self-quenching as the dye dilutes into the extravesicular medium. Experiments were done with the same lipid concentration (65 µM) as the FPE binding measurements and in buffer A so that data from the different experiments can be interpreted in a consistent manner. CF emission was measured at 520 nm (excitation at 490 nm). The fluorescence resulting from 100% release of encapsulated CF was determined by adding 10 µL of 20% Triton X-100. To ensure rapid mixing of peptide and vesicles and to avoid high local concentrations of peptide, 1 mL of a peptide solution at double the post-mix concentration was rapidly ejected from an Eppendorf pipette into 1 mL of a vesicle suspension at 130-µM concentration (65 µM post-mix) to initiate binding and dye release. The fluorescence emission intensity was measured 3 min after mixing CF-loaded vesicles with peptide. The dye release data were fitted to an equation defining a sigmoidal dependence on peptide concentration, yielding the *n* values listed in brackets in Table 2, where *f* is the fraction of total dye released, *f*_max_ is the maximum value of *f*, and *K* is a constant.

(4) $$f\;=\;f_{\text{max}}{\left[\text{peptide}\right]}^{n}/\left(K\;+\;{\left[\text{peptide}\right]}^{n}\right)$$

Circular dichroism (CD) spectra were measured at 20 °C using a Jobin-Yvon CD6 spectropolarimeter (Horiba Jobin Yvon, Longjumeau, France). All samples were made in buffer A. Vesicles for CD spectroscopy were extruded through 50-nm pore diameter filters to minimize the effects of light scattering, and spectra were measured in 0.1-mm path length cuvettes to further minimize scattering contributions. Spectra are averages of 9 scans with the appropriate peptide-free blank spectra subtracted and were zeroed at 260 nm before plotting without smoothing.

### 2.5 Outer membrane permeability

Outer and inner membrane permeabilities were determined as described previously [16]. *E. coli* cells overexpressing β-lactamase were grown to logarithmic phase, washed, and resuspended (10^8^ CFU/mL) in 10 mM HEPES buffer pH 7.4. About 90 µL of 1 × 10^7^ CFU of the test organism (*E. coli* 345-2) containing 20 µg/mL nitrocefin was then added to 96-well microtitre plates containing serial twofold dilutions of antimicrobial peptides. Nitrocefin cleavage was monitored by measuring *A*_490_. All data were measured in triplicate.

### 2.6 Inner membrane permeability

Inner membrane permeability was determined by measuring the β-galactosidase activity of lactose permease-deficient *E. coli* at 30 °C using ONPG as substrate. *E. coli* cells overexpressing the β- galactosidase enzyme (*E. coli* 122) were grown to logarithmic phase, washed, and resuspended (10^8^ CFU/mL) in phosphate buffered saline pH 7.4. About 90 µL of 1 × 10^7^ CFU of the test organism containing 0.4 mg/mL ONPG was then added to 96-well microtitre plates containing serial twofold dilutions of antimicrobial peptides. The production of *o-*nitrophenol was monitored spectrophotometrically at 420 nm. An equivalent volume of water replaced the peptide solution in the control assay. All data were measured in triplicate.

### 2.7 Peptide-induced bacterial lethality

MIC microtitre plates were set up as described before, and residual live bacteria following incubation at each peptide concentration were determined by plating aliquots onto Mueller Hinton agar plates. Plates were incubated at 37 °C, and the colony-forming units (CFUs) were counted after 24 hours. Peptide-induced lethality is expressed as a percentage of complete lethality, 100% indicating complete killing (no detectable colonies). All data were measured in triplicate.

## 3 Results

### 3.1 Role of the cysteine-15 residue in antimicrobial activity of RTA3

Each of the potential peptides in the *Streptococcus* *mitis* genetic element encoding RTA peptides has a Cys-15 residue^1^ (see amino acid sequence of RTA3 in Table 1), and preliminary (alanine scan) mutagenesis showed that replacement of Cys-15 with alanine greatly reduces antimicrobial activity (Table 1). These observations suggest a role for the C15 residue in high antimicrobial activity of RTA peptides.

To test the possibility that the C15 residue is involved in homo-dimerization, we attempted air oxidation of RTA3 using conditions that readily produce high disulfide yields in related Cys-containing antimicrobial peptides [17,18]. Surprisingly, no dimerization could be detected using mass spectrometry. The inability to homodimerize RTA3 (and the “wild-type” RTA1) is not due to structure formation in solution, since RTA peptides are unstructured at concentrations used for dimerization by air oxidation [1]. Indeed, chemical preactivation of RTA3 cysteine-thiols during synthesis was also unsuccessful in producing detectable dimerized peptide (Dr. G. Bloomberg, personal communication). We conclude that the sequence of bulky hydrophobic side chains in the C-terminus of the peptide precludes productive interactions of cysteine thiols on adjacent peptides. In addition, the antimicrobial activity of RTA3 was unaffected by the presence of 50 μM dithiothreitol (DTT) when tested against Gram-negative bacteria that are tolerant to DTT at this concentration (not shown). These observations support the conclusion that RTA3 is active as its free thiol form.

These observations do not rule out the possibility that hetero-disulfide formation involving the RTA3 C15 thiol and a thiol group in a protein in the target bacteria are important for activity, nor the possibility that ion chelation might have some role in antimicrobial activity, although the lack of effect of DTT on RTA3 activity does not support these interpretations. To explore the role of the C15 residue further, we prepared additional substitution variants, RTA3-C15S and RTA3-C15L, to investigate whether the C15 thiol could be replaced either with a hydroxyl group (serine) or with a residue sharing high hydrophobicity with cysteine (leucine). The antimicrobial activity of RTA3 was not recovered in the C15S mutant, but high activity was recovered in the RTA3-C15L replacement analogue, indicating that a dominant role of the C15 residue is to provide a bulky nonpolar side chain on the nonpolar face of the amphipathic helix (Table 1).

### 3.2 Peptide-induced membrane perturbation of vesicles containing trapped carboxyfluorescein

**Fig. 1:**
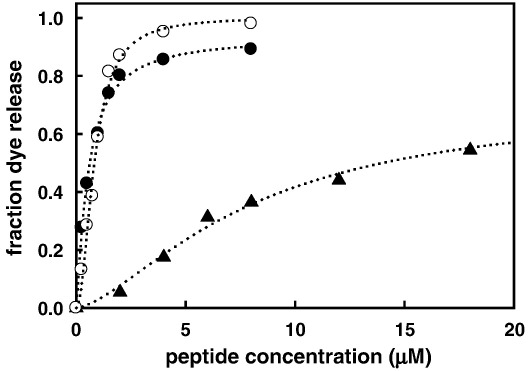
Peptide-induced CF release from 100 nm PE/PG/CL (80:10:15) LUV in buffer A. Peptides are (●) RTA3, (o) RTA3-C15L, (▲) RTA3-C15S. The total lipid concentration was 65 μM.

**Fig. 2:**
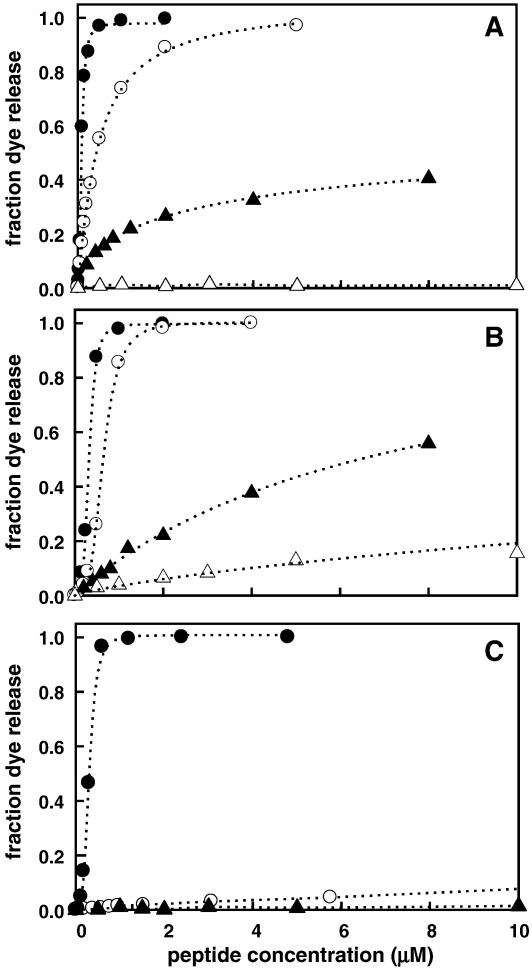
Peptide-induced CF release from 100 nm PC/PG LUV in buffer A, as a function of PG content. The total lipid concentration was 65 μM. Panels A, B, and C are data for RTA3, RTA3-C15L, and RTA3-C15S, respectively. Lipid mixtures are (●) PC/PG (50:50), (o) PC/PG (80:20), (▲) PC/PG (90:10), (Δ) PC (100%).

Previously, we showed that the ability of RTA3 peptides to bind at the surface of negatively charged phospholipid bilayer vesicles composed of 50% egg phosphatidylcholine (PC) and 50% egg phosphatidylglycerol (PG) and induce the release of trapped fluorescent dyes correlated well with antimicrobial activity against Gram-negative bacteria [1]. In the case of RTA3-C15S this correlation breaks down, so that while dye release from negatively charged membranes (PC/PG, 50:50) is reduced somewhat in RTA3-C15S compared to RTA3, the reduction is small (around 3-fold; see Fig. 2). However, when these experiments are repeated using a lipid composition more characteristic of Gram-negative bacterial membranes (PE/PG/CL, 80:10:15), RTA3-C15S becomes much less active (by around 15- to 20-fold) relative to RTA3 or RTA3-C15L (Fig. 1).

This observation may be interpreted in two ways. Either a specific property of the more complex membrane suppresses the ability of RTA3-C15S to bind and disrupt the membrane or this variant requires a high negative charge density to compensate for a reduction in amphipathic moment when a nonpolar amino acid (Cys or Leu) is replaced by a polar amino acid (Ser) on the nonpolar face of the amphipathic helix [19]. We tested this by measuring the relative membrane-perturbing activity of the peptides in a PC/PG mix in which the PG content was reduced from 50% to 20% or 10%. These data are shown in Fig. 2. RTA3-C15S is nearly as active as RTA3 and RTA3-C15L in permeabilizing membranes composed of 50% PG in PC, but when the negative membrane surface charge is suppressed by reducing the PG content to 20% (PC/PG, 80:20), RTA3-C15S is virtually inactive as a membrane permeabilizer (Fig. 2C). Both RTA3 and RTA3-C15L retain very strong membrane permeabilizing activity in mixed PC/PG bilayers containing only 20% PG (Figs. 2A and B). Only in 10% PG are the permeabilizing activities of RTA3 and RTA3-C15L substantially reduced. As previously shown [1], RTA3 is essentially inactive as a permeabilizer of pure PC bilayers, a consequence of the inability of the peptide to bind to a membrane lacking a negative surface charge. The slightly enhanced hydrophobicity of RTA3-C15L compared to RTA3 is probably the reason for the retention of weak permeabilizing activity in 100% PC bilayers (Fig. 2B).

### 3.3 Membrane binding of RTA3 peptide variants

Peptide-induced membrane permeabilization follows binding to the membrane surface. To assess the relationship between permeabilization and binding we measured binding of RTA3 and RTA3-C15S to vesicles composed of PC/PG (50:50) and PC/PG (80:20) using FPE (0.5%) as a binding-responsive fluorescent probe. In addition, we measured vesicle binding of variants containing a Phe9 to Trp9 substitution to monitor the blue shift in the Trp fluorescence emission spectrum that occurs upon membrane binding [1]. Like the Phe4 to Trp4 variants of RTA3 previously reported [1], the Phe9 to Trp9 variants have essentially the same antimicrobial activity as the unsubstituted peptides. Each of these analyses allows a quantitation of the change in binding free energy (Table 2) upon substituting Cys15 with Ser that can be used to interpret the amino acid specific contribution to binding in terms of published free energy scales (see Discussion).

**Fig. 3:**
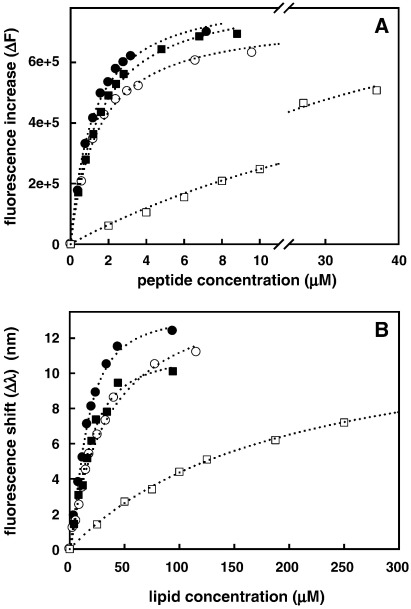
Binding of RTA3 peptides to PG/PC (50:50) (circles) and PG/PC (20:80) (squares). Panel A is RTA3 (filled symbols) and RTA3-C15S (open symbols) binding to 100 nm vesicles doped with 0.5% FPE in buffer A. The total lipid concentration was 65 μM. Dotted lines are fits to Eq. (2). Panel B is binding of RTA3-F9W (filled symbols) and RTA3-F9W,C15S (open circles) binding to undoped lipids in buffer A. Dotted lines in panel B are fits to Eq. (1).

**Fig. 4:**
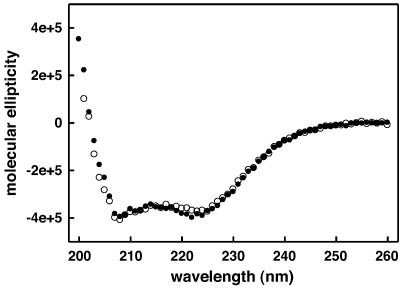
Circular dichroism spectra of RTA3 (●) and RTA3-C15S (o) in 50-nm diameter PC/PG (50:50) vesicles. Peptide and total vesicular lipid concentrations were 150 μM and 10 mM, respectively, in buffer A, and spectra were measured in 0.1-mm path length cuvettes at 20 °C.

Figs. 3A and B show binding of RTA3 and RTA3-C15S to FPE-doped vesicles [PC/PG (50:50) and PC/PG (80:20)] and RTA3-F9W and RTA3-F9W,C15S binding to undoped vesicles, respectively. In both cases, the C15S variants bound slightly more weakly to PC/PG bilayers containing 50% PG (by 1.3- to 2-fold) and the C15S variants bound much more weakly (by 9.3- to 12-fold) to bilayers containing only 20% PG. Since the peptides exhibit hyperbolic binding (Fig. 3), whereas dye release under our experimental protocol has a sigmoidal dependence on peptide concentration (Fig. 2; Table 2) [1], a small reduction in binding free energy to bilayers containing a reduced negative charge resulting from the Cys-15 to Ser substitution, translates into a large reduction in membrane disruption. RTA3 and RTA3-C15S have very similar CD spectra when bound to PC/PG (50:50) vesicles, indicating that the C15S substitution does not induce significant helix unwinding in the C15S variant when bound at the surface of the membrane with high negatively charged lipid content (Fig. 4).

These observations support the conclusions that the main effect of the serine substitution in RTA3-C15S in reducing antimicrobial activity, is *via* reduction of membrane binding, and that effects on target cell membranes are likely key elements in the antimicrobial activity of RTA3. We explore the latter conclusion below.

### 3.4 Membrane-selective effects of RTA3 peptides

To assess the relationships between membrane binding in model bilayers, membrane perturbation in bacterial membranes, and antimicrobial activity, we measured peptide-induced perturbation of inner (IM) and outer (OM) membranes of the Gram-negative bacterium *E. coli*. We compared the data from the RTA3 peptide variants with the clinically used antibiotic polymyxin E (colistin), a membrane active cyclic cationic peptide [20]. Polymixin E is known to act on the outer membrane of Gram-negative bacteria, interacting with lipopolysaccharide and increasing outer membrane permeability by displacing membrane-stabilizing divalent cations [20]. We tested the ability of RTA3 peptides and polymixin E to permeabilize the outer membrane (OM) and inner membrane (IM) of *E. coli* strains, *E coli* 345-2 and *E coli* 122, that overexpress perplasmic β-lactamase or cytoplasmic β-galactosidase, respectively. OM permeabilization promotes access of β-lactamase to the reporter substrate nitrocefin, and IM permeabilization promotes access of β-galactosidase to its reporter substrate ortho-nitrophenyl-β-galactoside (ONPG). In order to correlate membrane-specific permeabilization with antimicrobial activity, we also measured cell killing of the appropriate *E. coli* strains (*E. coli* 345-2 and *E. coli* 122) under the same conditions used for membrane permeability (but excluding reporter substrates).

**Fig. 5:**
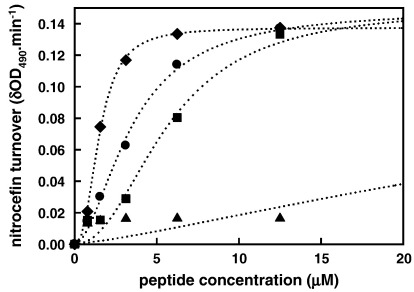
Peptide-induced permeabilization of the outer membrane of *E. coli* 345-2 to nitrocefin. Data in Mg^2+^-free medium is shown; see Table 3 for permeabilization data in the presence of 0.4 mM Mg^2+^. Peptides are (circles) RTA3, (squares) RTA3-C15L, (triangles) RTA3-C15S, and (diamonds) polymixin E.

**Fig. 6:**
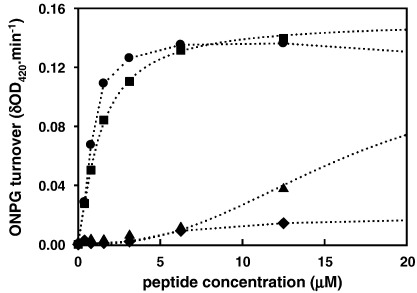
Peptide-induced permeabilization of the inner membrane of *E. coli* 122 to ONPG. Data in Mg^2+^-free medium is shown; see Table 3 for permeabilization data in the presence of 0.4 mM Mg^2+^. Peptides are (circles) RTA3, (squares) RTA3-C15L, (triangles) RTA3-C15S, and (diamonds) polymixin E.

**Fig. 7:**
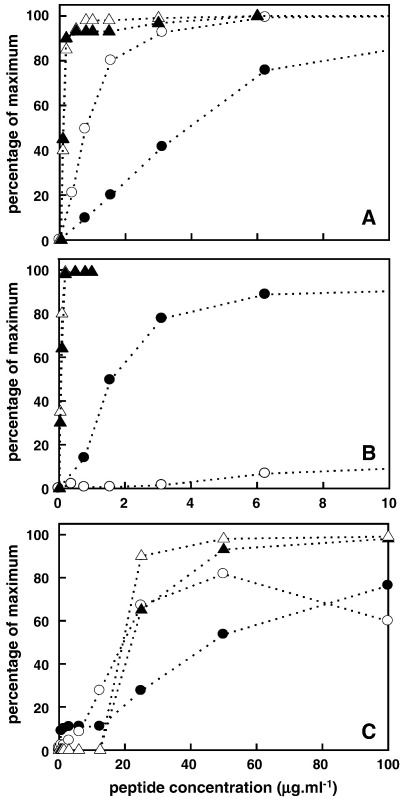
Peptide-induced permeabilization of *E coli* membranes and associated bacterial killing. Data are for RTA3 (A), polymixin E (B), and RTA3-C15S (C; note the expanded concentration scale). In each case, circles denote membrane permeabilization allowing access of marker substrates, and triangles denote bacterial killing as follows: (●) outer membrane permeabilization of *E. coli* 345-2; (o) inner membrane permeabilization of *E. coli* 122; (▲) killing of *E. coli* 345-2; (Δ) killing of *E. coli* 122.

These data are summarized in Figs. 5–7 and Table 3. We measured peptide-induced membrane-selective permeabilization in the absence and presence of 0.4 mM MgCl_2_ since divalent cations are known to stabilize Gram-negative bacterial membranes and are included in cation-adjusted Muller–Hinton media used to determine MICs. All of the peptides with high antimicrobial activities were effective in permeabilizing the outer membrane, allowing access of nitrocefin to periplasmic β-lactamase. RTA3-C15S, however, showed negligible OM permeabilizing activity (Fig. 5). Both RTA3 and polymixin E retain very high permeabilizing activity in the presence of 0.4 mM Mg^2+^ (Table 3), indicating an ability to compete with this divalent cation for binding sites on the outer membrane. The permeabilizing activity of RTA3-C15L was reduced somewhat by Mg^2^^+^, and this may be related to the reduced activity of this analogue in cation-adjusted Muller–Hinton media compared to RTA3 (Table 1). The low OM-permeabilizing activity of RTA3-C15S was abolished in the presence of Mg^2+^.

RTA3 and RTA3-C15L effectively permeabilize the inner membrane of *E coli* 122 allowing access of ONPG to cytoplasmic β-galactosidase (Fig. 6). The inactive RTA3-C15S is very poorly effective as an IM permeabilizer, as is polymixin E, consistent with previous observations using polymixins (polymixin B) [21]. The permeabilizing activities of both RTA3 and RTA3-C15L are reduced somewhat by 0.4 mM Mg^2+^. It is notable that the membrane permeabilizing activities of RTA3 and RTA3-C15L on the IM and OM in Mg^2+^-containing media are comparable to the MICs of these peptide against *E. coli* strains measured under standard conditions in cation-adjusted Muller–Hinton media (Table 1).

When membrane-selective permeabilization is compared with bacterial cell killing under equivalent conditions (Fig. 7), the relationships between membrane permeabilization and cell killing are more apparent. RTA3 effectively kills *E.* *coli* 345-2 at concentrations well below those yielding significant OM permeabilization to nitrocefin, whereas concentrations that kill *E.* *coli* 122 are closer to (but still lower than) those permeabilizing the IM (Fig. 7A). On the other hand, polymixin E kills *E. coli* 122 at concentrations well below those required for IM permeabilization that allows ONPG access to the cytoplasm. As expected, the poor membrane-permeabilizing activity of RTA3-C15S is matched by very low bacterial cell killing activity unless peptide concentrations are considerably enhanced (Fig. 7C; note the expanded concentration scale on this panel). In this case, cell killing and membrane permeabilization to IM and OM marker substrates occur at equivalent peptide concentrations, indicating a closer association of membrane permeabilization (especially IM permeabilization) and bacterial killing.

## 4 Discussion

The data presented indicate that the Cys-15 residue in RTA3 is not absolutely required for high antimicrobial activity, since the cysteine can be replaced with a leucine with the retention of much of the activity of the native peptide. We have employed these peptides, as well as an RTA3-C15S variant, to address mechanistic aspects of the anti-Gram-negative activity of RTA3.

### 4.1 Surface charge effects on RTA3-C15S membrane binding

Comparison of RTA3 and RTA3-C15S highlights the interplay of amphipathicity and electrostatics that underlies the combination of high antimicrobial activity and strong prokaryotic-selective toxicity required for therapeutic potential in positively charged helical antimicrobial peptides. While RTA3-C15S has antimicrobial activity reduced by around 60-fold relative to RTA3 (Table 1), RTA3-C15S is only 2- to 3-fold less effective than RTA3 in disrupting membranes composed of equimolar PC and PG (Fig. 2). However, if these peptides are tested for their abilities to permeabilize vesicles composed of lipids having negative charge density closer to that of the inner membrane (IM) of Gram-negative bacteria (i.e., PE/PG/CL, 80:10:15 or PC/PG, 80:20; Figs. 1 and 2), the permeabilizing activity of RTA3-C15S is greatly reduced (up to 100-fold) while RTA3 and RTA3-C15L retain very high activity. Both the absolute and relative effectiveness in killing Gram-negative bacteria (*E. coli* and *P.* *aeruginosa*) are broadly reproduced in PC bilayers containing 20% PG.

**Fig. 8:**
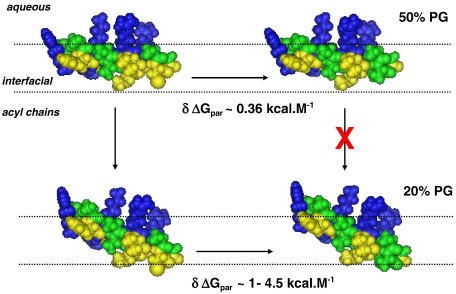
Schematic illustration of the effect of the RTA3-C15S mutation on interfacial membrane binding according to the depth of membrane surface penetration. The small (1.3- to 2-fold; δΔ*G* ∼ 0.2–0.4 kcal/mol) reduction in membrane binding for RTA3-C15S (right) compared to RTA3 (left) in PC/PG (50:50) bilayers (Fig. 3; Table 2) is consistent with an interfacial localization of the C15 residue, since the free energy “cost” of a Cys-Ser replacement is around 0.36 kcal/mol according to the interfacial partitioning scale of Wimley and White [22]. A more dramatic loss of membrane partitioning is expected for this mutation if the C-terminal region lies somewhat deeper in the bilayer in a PC/PG (80:20) membrane. In this case, the free energy “cost” is expected to be in the range of 1–4.5 kcal/mol based on the Hessa [24] and Wolfenden [23] scales for partitioning of amino acid side chains towards the nonpolar regions of the membrane, consistent with the reduced binding constant (9.5- to 12-fold; δΔ*G* ∼ 1.3–1.4 kcal/mol) of RTA3-C15S compared to RTA3 in 20% PG bilayers (Fig. 3; Table 2). Hydrophobic amino acids are colored yellow; polar uncharged residues are green; Lys, Arg residues are blue.

The origin of the large selective loss of membrane-permeabilizing activity in RTA3-C15S that results from reducing the PG content of mixed PC/PG bilayers from 50% to 20% can be assessed by comparison of lipid binding affinities. The ratios of binding constants of RTA3 and RTA3-C15S in 50% PC/PG [1.4:1 (from FPE fluorescence) and 2.0:1 (Trp fluorescence)] and in 80% PC/20% PG [9.3:1 (FPE) and 12:1(Trp)] are similar to those expected if the context of the C15→S substitution in 50% PG bilayers is the membrane interfacial region [22], whereas the context of the same substitution in 20% PG is a somewhat deeper region of the bilayer where the environment is more hydrophobic [23,24]. This is illustrated in the schematic of Fig. 8 which shows space-filling models of RTA3 and RTA3-C15S in a helical conformation ([1]; Fig. 4), at the membrane interface. Interfacially localized helical peptides lie at a shallow depth in the bilayer [25], and this is likely to be promoted by high negative charge density for positively charged peptides. The free energy cost of replacing an interfacially located cysteine residue with a serine is small (∼ 0.37 kcal/mol), according to the free energy scale of Wimley and White for interfacial partitioning in POPC [22], similar to that found for the RTA3-C15S substitution in PC/PG (50:50) (∼ 0.2–0.4 kcal/mol; Table 2). In a membrane with reduced negative charge density, the reduction in the electrostatic contribution to binding may be compensated in RTA3 by sinking of the peptide slightly deeper into the bilayer (or tilting of the helix axis with respect to the membrane surface) such that the bulky hydrophobic side chains that line the hydrophobic face of the helix near the C-terminus make productive interactions with the acyl chain region of the bilayer (Fig. 8). In this context, replacing a cysteine side chain with a serine side chain can “cost” from between 1 kcal/mol to 4.68 kcal/mol, based, respectively, on the preference of amino acid side chains to fall within the transmembrane regions of membrane-spanning peptides [24] and the transfer of amino acid side chains from water to cyclohexane [23]. Since the water–cyclohexane partitioning scale for amino acid side chains is an overextreme measure of the hydrophobicity of a membrane environment in the context of a surface-lying amphipathic helical peptide, an intermediate free energy cost, equivalent to a reduced surface partitioning of RTA3-C15S compared to RTA3 near 9.3- to 12-fold (δΔ*G* ∼ 1.3–1.4 kcal/mol; Table 2), is consistent with a small movement of the C-terminal region of the peptide towards the less polar regions of the bilayer. We note that a strict quantitative interpretation of this data in terms of the interfacial partitioning free energy scale requires that the *difference* in interfacial partition free energies (Cys→Ser) is the same in unsaturated PC membranes containing 50% or 20% PG (this study), as in POPC [22]. While the membrane composition dependence of interfacial partitioning free energies has not yet been systematically explored, this assumption is likely to be most reliable for amino acid substitutions involving uncharged amino acids. The observation that the C15S substitution has a very small effect (1.5- to 2-fold reduction) on interfacial partitioning of RTA3 in PC/PG (50:50), and a much larger effect (9.3- to 12-fold) in PC/PG (80:20) is independent of any assumptions in the use of free energy scales for analyzing membrane binding.

The membrane perturbations induced by amphipathic peptides in vesicles often show sigmoidal concentration dependence, and as indicated by peptide cross-linking experiments [18,26], this can indicate cooperativity in the peptide-induced membrane disruption that underlies efflux of vesicle-entrapped molecules. Without similarly establishing cooperativity in the mechanism of peptide-induced membrane permeabilization, cooperativity (e.g., pore formation involving self-association of membrane-bound peptide) cannot be assumed from a sigmoidal concentration dependence, especially when using an experimental protocol in which the kinetics of dye release is sampled at a single time point. However, a combination of hyperbolic (noncooperative) membrane binding and a sigmoidal peptide concentration dependence for dye release underlies the observation that relatively small (∼ 10-fold) reductions in binding affinities can result in much larger reductions (∼ 50-100-fold) in membrane perturbation. These subtle differences in membrane binding to membranes with different negative charge densities may underlie some of the variation in peptide antimicrobial activities against different bacterial species [2–7,27]. Detailed analysis of the thermodynamics of membrane binding may allow the relationships between peptide sequence variation and organism- and strain-specific antimicrobial activities to be understood in more detail.

### 4.2 Membrane-specific effects of RTA3 peptides on bacterial cells

The general relationship between antimicrobial activity against Gram-negative bacteria and the permeabilization of negatively charged membranes having reduced negative charge density (Figs. 1 and 2; Tables 1 and 2; see also [1]) supports a significant role for membrane activity in the antimicrobial mechanism(s) of these peptides. The effects of the peptides in permeabilizing *E. coli* inner and outer membranes highlight differences in the properties of RTA3 peptides and polymixin E (consistent with the observation of RTA3–polymixin E synergies).^1^ Both polymixin E and active RTA3 variants (RTA3; RTA3-C15L) strongly permeabilize the OM to nitrocefin whereas only the RTA3 peptides permeabilize the IM to ONPG.

Polymixin E is known to bind to lipopolysaccharide on the OM, permeabilizing the OM and promoting self-uptake of the peptide into the periplasm [20]. Direct comparison of bacterial cell killing under conditions equivalent to the permeabilization assays indicates that all of the active peptides kill *E. coli* strains at concentrations below those required for outer or inner membrane permeabilization to the marker substrates nitrocefin or ONPG. This is particularly marked for polymixin E, where cell killing and inner membrane permeabilization to ONPG are completely dissociated (Fig. 7B). Previous studies demonstrate that polymixin B can permeabilize the IM to ions at peptide concentrations that are closer to the MICs [21], and the similarities between polymixins B and E suggest that the killing mechanism of polymixin E may involve effects on the IM [28]. However, the observations demonstrate that polymixin E does not induce marked IM permeability to molecules of the size of ONPG in the manner of the active RTA3 peptides.

The IM permeabilization to ONPG by active RTA3 peptides occurs at concentrations slightly higher than those required for cell killing; in the case of the poorly active RTA3-C15S, cell killing is induced only at concentrations that promote permeabilization of the IM. These observations are consistent with the conclusion that IM perturbation by active RTA3 peptides at concentrations slightly below those required to allow permeabilization to ONPG may be associated with target cell death either by direct effects on membrane functions [29], permeabilization to molecules (water and ions) smaller than ONPG (MW 301), or following translocation of peptide into the bacterial cytoplasm to act on intracellular targets [30,31].

The results presented support the conclusion that bacterial cell killing by RTA3 peptides derived from a commensal organism is associated with bacterial membrane perturbation and that these properties can be understood in relation to the thermodynamics of membrane binding to lipids in which the negative surface charge is matched to that of the target cell membrane; for RTA3 peptides, membranes composed of unsaturated 20% PG/80% PC lipids provide a good analogue for peptide activity against Gram-negative bacteria *E. coli* and *P. aeruginosa*. Subtle changes in amino acid composition that have small effects on membrane binding can have large effects on membrane perturbation because of the apparent cooperativity in membrane perturbation, and these effects seem to be reflected in the relative activities against target bacteria. An extended analysis of the membrane binding of substitution analogues of peptides of this class is likely to be useful in further testing and refining free energy scales for amino acid contributions to membrane partitioning of polypeptides, especially in narrowing the wide range of free energies associated with deeper insertion of amino acids into membranes [23,24,32] that likely relates to structural and sequence context.

## Figures and Tables

**Table 1:** Biological activities of peptides.

	MIC			
	*P. aeruginosa*	*E. coli*	*E. coli*	*E. coli*
	ATCC 27853	ATCC 25922	UB 122	345-2
	µM	µM	µM	µM
RTA3^a^	4	2	4	4
RTA3-C15S	275	138	138	69
RTA3-C15A	277	139	138	70
RTA3-C15L	17	8	4	4
Polymyxin E	2	1	0.5	0.5

a RTA3 amino acid sequence is: RPAFRKAAFRVMRACV-NH_2_.

**Table 2:** Effects of Cys_15_-Ser substitution on membrane interactions of RTA3.

	Binding		CF dye-release^a^	
	50% PG	20% PG	50% PG	20% PG
	FPE fluorescence^b^		μM	μM
	μM	μM		
RTA3	1.2	1.5	0.09 (2.3^c^)	0.40 (1.7)
RTA3-C15S	1.6	18	0.30 (2.5)	> 50 (ND)
δ*ΔG*_C15->S_ (kcal/mol)^d^	0.18	1.4		

	Trp fluorescence^e^			
	μM	μM		

RTA3-F9W	15	16		
RTA3-F9W,C15S	31	148		
δΔ*G*_C15->S_ (kcal/mol)	0.42	1.3		

a Peptide concentration that induced half-maximal CF dye release from 100 nm LUV (65 μM total lipid).

b Peptide concentration that induced half-maximal fluorescence shift in FPE-doped vesicles.

c Value of *n* (peptide concentration power dependence) in the sigmoidal fit of dye release data to Eq. (4).

d Free energy contribution to membrane binding arising from the C15 to S substitution.

e Lipid concentration (as 100 nm LUV) that induced 50% peptide binding; peptide concentration was 2 μM.

**Table 3:** Peptide permeabilization of *E. coli* Outer and Inner membrane.

	OM		IM	
	−Mg^2+^	+ Mg^2+^	−Mg^2+^	+ Mg^2^
	μM	μM	μM	μM
RTA3	3^a^	3.5	0.8	2.5
RTA3-C15S	> 70	NA^b^	18	> 100
RTA3-C15L	6	11	1	6
Polymixin E	2	1.8	> 40	> 100

a Values are the peptide concentration resulting in half-maximal translocated substrate turnover.

b No activity (no measurable permeabilization at 50 μM peptide concentration).

## Footnotes

1 A. Hawrani, C. E. Dempsey, R. A. Howe, and T. R. Walsh, presented at the 47th ICAAC meeting, Chicago, September 2007.
